# Seroepidemiologic Screening for Zoonotic Viral Infections, Maputo, Mozambique

**DOI:** 10.3201/eid2205.151002

**Published:** 2016-05

**Authors:** Eduardo Samo Gudo, Birgitta Lesko, Sirkka Vene, Nina Lagerqvist, Sandra Isabel Candido, Nilsa Razão de Deus, Félix Dinis Pinto, Gabriela Pinto, Vanessa Monteiro, Virginia Lara Evaristo, Nilesh Bhatt, Ivan Manhica, Kerstin I. Falk

**Affiliations:** Instituto Nacional de Saúde, Ministry of Health, Maputo, Mozambique (E. Samo Gudo, S.I. Candido, N.R. de Deus, F. Pinto, G. Pinto, V. Monteiro, V.L. Evaristo, N. Bhatt);; Public Health Agency of Sweden, Solna, Sweden (B. Lesko, S. Vene, N. Lagerqvist, K.I. Falk);; Karolinska Institutet, Stockholm, Sweden (B. Lesko, K.I. Falk);; National Directorate for Public Health, Ministry of Health, Mozambique (I. Manhica)

**Keywords:** zoonoses, arbovirus infections, seroepidemiologic study, seroprevalence, Mozambique, vector-borne infections, viruses

**To the Editor:** In sub-Saharan Africa, febrile patients are often assumed to have, and are treated for, malaria, but when tested, many are malaria-negative. Because emerging diseases, such as chikungunya virus (CHIKV) and dengue virus (DENV) infections, cause outbreaks around the world (*1*–*3*), the importance of these pathogens has become more evident. However, low-income countries have limited epidemiologic data on alternative diagnoses to malaria (*4*,*5*) and poor laboratory capacity (*1*), which restrict further diagnostic investigations. An early study in Mozambique during the 1980s found antibodies to Rift Valley fever virus (RVFV) in 2% of pregnant women (*6*). More recently, a RVFV seroprevalence of 36.9% among cattle in the Maputo Province was shown in 2010–2011 (*7*). Furthermore, the movement of humans from rural areas to major cities, particularly to the capital of Maputo, might affect human illnesses and disease pattern of zoonotic viruses (*3*).

We conducted a pilot study on CHIKV, DENV, hantavirus, RVFV, and West Nile virus (WNV) epidemiology in Mozambique. Ethical approval (registration no. IRB00002657) was granted by the National Bioethics Committee in Mozambique and by the Regional Ethical Review Board at Karolinska Institutet, Stockholm, Sweden (permit no. 2012/974–31/3).

During 2012–2013, a total of 78 febrile patients were prospectively enrolled when they sought medical attention at the Polana Caniço Health Center and Mavalane Health Center (catchment area 4,663 km^2^, estimated population 46,184 inhabitants) in the suburban area of Maputo city. All included patients answered a questionnaire and were initially screened for malaria by blood smear light microscopy; 15 were positive for malaria (Table). Patients’ median age was 29 years (37 years for seropositive patients) and ranged from 5 to 78 years. Forty-six (59%) were female. Fifty-eight (74%) reported recent exposure to mosquitoes. None of these persons had a history of international travel, and none had received a yellow fever vaccination.

**Table:** Results of screening for viral antibodies and malaria parasites in 78 febrile patients, Maputo, Mozambique, 2012–2013*

Organism	No. (%) positive†
Chikungunya virus	15 (19.2)
Dengue virus	10 (12.8)‡
Hantavirus	0
Rift Valley fever virus	1 (1.3)
West Nile virus	3 (3.8)
Malaria parasites	15 (19.2)§

*Viral antibody–positive patients had positive IgG or IgM response for >1 of the zoonotic viruses in acute- or convalescent-phase serum samples. The overall malaria screening results for the study cohort is also presented †Three of the 23 serology-positive patients were positive for dengue virus and West Nile virus IgG, of whom 2 were also positive for chikungunya virus IgG and 1 for Rift Valley fever virus IgG. ‡Including 2 dengue virus IgM-positive samples. §Three of 15 malaria-positive patients had a positive serologic finding (2 for dengue virus IgG and 1 for chikungunya IgG).

Sixty (77%) patients provided paired acute- and convalescent-phase blood samples, with a minimum of 14 days (median 33 days) between samples. Serum samples were sent to the Public Health Agency of Sweden and blindly screened at a titer of 1:20 for IgG to CHIKV, DENV, hantavirus, RVFV, and WNV by using in-house indirect immunofluorescence assays as described for DENV by Vene et al. (*8*). Screening for IgG was done on convalescent-phase serum samples or, when those were not available, on acute-phase serum samples. Further immunofluorescence analyses for titer increases were performed for patients for whom paired serum samples were available and screening results were positive for IgG; however, no titer increases were found. Serum from admittance were tested for DENV IgM and WNV IgM by using commercial assays according to manufacturers’ instructions (Panbio Dengue IgM Capture ELISA E-DEN01M/E-DEN01M05, Standard Diagnostics, Inc., Yongin-si, South Korea; Serion ELISA classic ESR14M West Nile Virus IgM, Institut Virion/Serion GmbH, Würzburg, Germany); 2 samples were positive for DENV IgM but none for WNV IgM. All acute serum samples were screened by using 1-step real-time reverse transcription PCR for CHIKV, RVFV, WNV (in-house validated assays), and DENV (*9*). Results were negative for viral RNA.

Twenty-three (29%) of the 78 patients had a positive serology result from acute- or convalescent-phase serum samples for >1 of the tested viral pathogens (Table). The main finding was CHIKV IgG in 15 (19%) patients. Ten (13%) patients had positive results for DENV, including 2 DENV IgM–positive samples.

The seroepidemiologic findings in this pilot study in Maputo strongly suggest possible and neglected alternative causes of febrile illness in Mozambique. Antibodies to CHIKV were found in 19% of the patients, which was a novel finding for Mozambique but corresponded well with other reports on the spread of CHIKV in tropical and subtropical areas of the world (*2*,*3*). DENV antibodies were present in 13% of the study population, representing a new finding in southern Mozambique; previous outbreaks have been reported from the northern part of the country (*5*). The median age of the seropositive patients (37 years) was higher than for the group as a whole (29 years), which might reflect increased exposure to zoonotic viruses over time. One patient was IgG positive for RVFV, a potentially emerging cause of fever in Mozambique, especially in view of recent reports of RVFV in cattle (*7*). The samples positive for both DENV and WNV IgG could represent previous independent infections with these viruses, co-infection, or cross-reactivity, which are common for flavivirus IgG (*10*). 

Overall, results indicate that exposure to vectorborne viruses in persons living in suburban areas of Maputo city is frequent, suggesting that infections with CHIKV, DENV, and RVFV infection should be considered as alternative diagnoses for patients with febrile illness in these settings. On the basis of these results, more extensive research is planned on the epidemiology of zoonotic viral infections in Mozambique.
